# The Challenge of Dual Diagnosis

**Published:** 1996

**Authors:** George Woody

**Affiliations:** George Woody, M.D., is a clinical professor of psychiatry at the University of Pennsylvania and chief of the substance abuse treatment unit at the Philadelphia Veterans Affairs Medical Center

**Keywords:** dual diagnosis, AOD dependence, comorbidity, behavioral and mental disorder, diagnostic criteria, prevalence, etiology, diagnosis, health care delivery, treatment program, treatment outcome

## Abstract

Researchers have made great strides in understanding and treating alcoholics with co-occurring psychiatric disorders. Improved diagnostic criteria are available, and research has demonstrated that both disorders must be addressed if the dually diagnosed patient is to have the best chance for a good outcome. The best type of treatment program is an integrated approach, assuring that treatments will be coordinated for best effect. Additional research is needed to match optimum treatment approaches with cost-effective reimbursement practices.

The traditional view that psychiatric disorders are unrelated to alcohol and other drug (AOD)-use problems^1^ has hampered effective treatment of patients who exhibit both types of disorder (Ries 1993; Miller 1994). Psychiatric and AOD disorders produce many similar symptoms and often coexist in the same patient, where one disorder can influence the course and treatment outcome of the other. The existence of two or more different disorders in the same patient is referred to as comorbidity. Patients with comorbid AOD and psychiatric disorders are said to have dual disorders, or a dual diagnosis.^2^

Research indicates that patients with dual diagnoses are more disabled and require more services than patients with a single disorder. In addition, they are more prone to suicide (Cornelius et al. 1995) and have higher rates of homelessness and legal and medical problems as well as more frequent and longer hospitalizations (see Center for Substance Abuse Treatment [CSAT] 1994).

Patients with dual disorders may be misdiagnosed and improperly treated, often “falling through the cracks” in the health care system (Merikangas and Gelernter 1990; Minkoff 1989; Ries 1993). For example, alcoholics with psychiatric disorders may be rejected by both alcoholism programs and mental health programs (National Institute on Alcohol Abuse and Alcoholism 1991). This article explores some problems in diagnosing and treating alcoholics with dual diagnoses.

## How Common Is Dual Diagnosis?

Two large epidemiologic studies have provided data on the prevalence of dual diagnosis in the general population. The Epidemiologic Catchment Area (ECA) study sought data on psychiatric disorders and their treatment from more than 20,000 residents of households, group homes, and long-term institutions in five sites across the United States (Regier et al. 1990). The ECA found that 13.5 percent of respondents had experienced an alcohol-use disorder at some time in their lives, 6.1 percent had experienced other drug-use disorders, and 22.5 percent had experienced non-AOD psychiatric disorders (Regier et al. 1990). Lifetime prevalence for any psychiatric or AOD disorder was 34 percent (Helzer and Pryzbeck 1988). Overall, the lifetime prevalence for any psychiatric disorder was 44 percent among people with an alcohol disorder and 64.4 percent among people with other drug-use disorders (Regier et al. 1990).

More recently, the National Comorbidity Study (NCS) administered structured psychiatric interviews to more than 8,000 respondents ages 15 to 54 in the noninstitutionalized civilian population of the United States. The NCS found higher rates than the ECA for any or all lifetime disorders (i.e., 48 percent) (Kessler et al. 1996). As in the ECA, the NCS found most mental disorders to be more common among persons with a current or lifetime AOD diagnosis than among those who had never experienced AOD problems (Kessler et al. 1996).

The NCS also found that most disorders had their onset prior to the onset of the AOD disorder. A significant exception to this general finding was mood disorders (e.g., depression) among male alcoholics, which usually developed after the onset of the alcoholism (Kessler et al. 1996).

## Psychiatric Problems Associated With AOD Disorders

Dually diagnosed patients most often exhibit symptoms of an apparent mood disorder that can range from dysthymia to a major depressive episode. Symptoms of anxiety are also a common feature, often mixed with symptoms of depression. Disorders that involve disturbances in thinking, such as mania and schizophrenia, occur less frequently.

As discussed below, the occurrence of isolated psychiatric symptoms, however severe, does not always justify the diagnosis of an independent psychiatric disorder. Nevertheless, ECA data indicate that alcoholics are also 21.0 times more likely to have a diagnosis of antisocial personality disorder compared with nonalcoholics.^3^ Similar statistics (i.e., odds ratios) include 3.9 times for drug abuse; 6.2 times for mania; and 4.0 times for schizophrenia. Despite the association of symptoms of depression and anxiety with alcoholism, this survey found only a mild increase in major depressive disorder and essentially no increase in anxiety disorders in alcoholics compared with nonalcoholics (Helzer and Pryzbeck 1988).

## What Accounts for Dual Diagnosis?

The extensive association between alcoholism and psychiatric disorders does not directly support any conclusions about causality. Any of various factors might contribute to dual diagnosis, including the following (Schuckit 1986; Meyer 1989): (1) alcoholism and a psychiatric disorder can co-occur, either sequentially or simultaneously, by coincidence; (2) alcoholism can cause certain psychiatric conditions or increase their severity; (3) psychiatric disorders might cause alcoholism or increase its severity; (4) both alcoholism and a psychiatric disorder may be caused separately by some third condition; (5) alcohol use or alcohol withdrawal can produce symptoms that mimic those of an independent psychiatric disorder.

The development of these concepts has advanced our understanding of dual diagnosis. Earlier schools of thought about possible causal relations of psychiatric and AOD disorders approached opposite ends of a continuum based on the differing perspectives of addiction and psychiatric professionals (Schuckit 1985).

Many alcoholism researchers and clinicians have expressed the view that all or most comorbid psychiatric problems are produced by alcohol use and are therefore secondary to the alcoholism. In this view, adequate treatment of alcoholism is sufficient to resolve co-occurring psychiatric problems, and additional psychiatric treatment is usually unnecessary. At its extreme, this view has resulted in alcoholics being advised at self-help group meetings to discontinue essential psychiatric medications (Woody et al. 1995).

At the other end of the continuum is the view that alcoholism may develop when people take drugs to self-medicate symptoms of a preexisting psychiatric disorder. This hypothesis implies that treatment of the psychiatric problem is necessary and even sufficient for treatment of the alcoholism. In extreme cases, clinicians have treated some dually diagnosed patients psychiatrically for years without making any effort to address the patients’ alcoholism directly (Woody et al. 1995; Miller 1994).

### Toward an Integrated View

Most current data indicate that each of the above views may be true, to a greater or lesser extent, in different patients. Extensive research indicates that alcohol use can produce psychiatric symptoms or exacerbate existing ones (McLellan et al. 1979; Schuckit 1983; Schuckit and Monteiro 1988). This finding is especially clear in the case of depression and anxiety produced by alcohol consumption or withdrawal.^4^ Alcohol-induced psychiatric symptoms decrease with abstinence, providing evidence that they are not independent disorders (Kadden et al. 1995).

In addition, alcoholics undergoing prolonged periods of alternating intoxication and withdrawal often exhibit symptoms such as hallucinations and thought disturbances. Although these symptoms suggest schizophrenia or mania, they can be induced by alcohol consumption in the absence of an independent psychiatric disorder (Miller 1994).

With respect to self-medication, many case reports and much clinical experience indicate that some patients use alcohol to reduce the intensity of the anxiety, tension, depressed mood, insomnia, apathy, and social isolation associated with independent mental disorders (Khantzian 1985; Goodwin and Jamison 1990). Perhaps because of impaired perception or lack of insight, mentally disordered persons may persist in such use despite long-term alcohol-induced *worsening* of their symptoms (Winokur et al. 1995). Whether use of alcohol for self-medication can develop into true alcoholism in susceptible people is a matter of debate (Raskin and Miller 1993; Winokur et al. 1995).^5^

The existence of multiple paths to the development of psychiatric symptoms highlights the importance of patient diversity (i.e., heterogeneity) and the need for individualized assessments (Rounsaville et al. 1983). Epidemiologic studies group subjects according to their similarities and help minimize the effects of rationalization and denial (Hesselbrock et al. 1986). However, awareness of the multiplicity of genetic, psychosocial, and other factors is important to the diagnosis and treatment of the individual patient (Roy et al. 1991).

## Diagnosis

Current advances in diagnosis include the use of structured interviews, specific descriptions of alcohol-induced mental disorders, and guidelines for differentiating alcohol-induced from primary mental disorders (i.e., the *Diagnostic and Statistic Manual of Mental Disorders, Fourth Edition* [DSM–IV]). These advances are contributing to the development of more complex models and a better understanding of these disorders.

Effective treatment of dual disorders begins with a thorough diagnostic assessment. The frequent occurrence of psychiatric or addictive symptoms in the absence of an independent disorder, as discussed previously, suggests the importance of distinguishing between drinking and alcoholism; sadness and depression; and anxiety feelings and major anxiety disorders (Schuckit and Monteiro 1988). Many mistakes can be avoided by the careful use of appropriate diagnostic criteria.

Structured interviews have been shown to be the most reliable diagnostic instruments. Among these are the Structured Clinical Interview for the *Diagnostic and Statistical Manual of Mental Disorders, Third Edition, Revised* (SCID); the Composite International Diagnostic Interview (CIDI); and the Diagnostic Interview Schedule (DIS) (for review, see Grant and Towle 1990 and Allen and Columbus 1995). Efforts are under way to modify these instruments according to DSM–IV guidelines.

### DSM–IV Guidelines

The DSM–IV (American Psychiatric Association [APA] 1994) is a standard guide to defining and diagnosing psychiatric and addictive disorders. In many cases, the DSM–IV provides exclusionary criteria to help distinguish between AOD-induced symptoms and independent disorders.

According to the DSM–IV guidelines, psychopathology should be labeled AOD-induced if (1) it occurs only during periods of intoxication or withdrawal, (2) the symptoms are consistent with those of the particular AOD’s that the patient is using, and (3) the symptoms are not better accounted for by another disorder. Conversely, a psychiatric problem should be labeled a primary, non-AOD-induced disorder if it (1) developed prior to the AOD use; (2) has been present during periods of abstinence extending beyond 1 month; (3) has symptoms that are not consistent with those produced by the AOD’s; or if (4) the psychiatric symptoms are better accounted for by a non-AOD-induced disorder, such as a medical condition (APA 1994).

## Treatment

Because each dual disorder can aggravate the course of the other, both disorders must be treated if the patient is to have the best chance for a good outcome (Woody et al. 1995). The first step in treatment is to perform an accurate diagnosis. The treatments recommended are similar to the treatments effective for the individual disorders (Woody et al. 1995). For example, a patient with alcoholism and mania needs alcoholism treatment that may involve detoxification followed by alcohol-focused therapy and participation in a self-help group. In addition, the patient must simultaneously receive ongoing psychiatric treatment with appropriate antimanic medication (e.g., lithium).

All these treatments can be provided by a single clinician trained in both approaches or by a team of specialists. For example, alcoholism therapy is administered individually or in a group setting by one or more alcoholism counselors, whereas the psychiatric treatment (including counseling and medications management) is administered by a psychiatrist. The main requirement for a successful outcome is that the treatments be *coordinated*. In addition, the treatments are usually most effective when delivered in the same setting, because that arrangement fosters good communication between members of the treatment team and is most convenient for the patient.

Unfortunately, this kind of coordinated care is often unavailable. Arbitrary and historically based separations exist between the mental health and alcoholism treatment systems. Traditionally, each system treats only one kind of disorder; consequently, the patient must enroll in separate programs to achieve total care (Green 1996). This poses special problems for the dually diagnosed, who tend to have difficulty organizing their affairs and who may lack the means of transportation between facilities.

Special dual diagnosis programs have been developed to address this problem. Many of these programs have been established within inpatient psychiatric units, resulting in high costs that may not be authorized by managed-care organizations. Outpatient programs are less expensive and can be highly effective if they are appropriately staffed and backed up by inpatient services for emergencies.

Currently, even outpatient programs have become subject to cost cutting, often constraining providers to focus on treating only one of the two dual disorders (Kessler 1996). This practice undermines the concept of integrated treatment and may eventually result in even higher costs through the increased need for expensive followup emergency and inpatient services.

### Delivery of Treatment

Three general approaches are used in delivering treatment to the dually diagnosed patient. One approach is to treat one disorder first and then the other (i.e., sequential treatment); the second approach is to treat both disorders simultaneously but in different settings (i.e., parallel treatment); the third is to treat both disorders simultaneously in the same setting (i.e., integrated treatment). Historically the most common approach to dual diagnosis has been sequential treatment. Some clinicians believe that addiction treatment must be administered first, so that the patient can be in stable recovery before entering psychiatric treatment. Other clinicians believe that psychiatric treatment should be administered first. Still other clinicians believe that the relative severity of the patient’s addictive or psychiatric symptoms should determine sequence of treatment or that the disorder that appeared first should be treated first (Miller 1994).

In practice, treatment sequence should vary depending on the situation. For example, psychiatric problems require immediate attention among patients exhibiting acute episodes of a major psychiatric disorder, whether alcohol-related or independent. Examples include schizophrenia, mania, AOD-induced psychoses, or AOD-induced depression with suicidal behavior. In other cases, the AOD disorder tends to be treated first (Woody et al. 1995).

In the parallel approach (Miller 1994), treatment for both disorders is administered simultaneously, although generally at different facilities. For example, a patient may participate in AOD education and drug refusal classes at an addiction center; participate in a self-help group, such as Alcoholics Anonymous; and attend group therapy and medication education classes at a mental health center. Both parallel and sequential treatment utilize existing treatment programs and settings. Thus, mental health treatment is provided by mental health clinicians, and addiction treatment is provided by addiction treatment clinicians. Coordination between settings is variable, and patients may receive conflicting explanations and advice. Sequential and parallel treatment may be most appropriate for patients who have a very severe problem with one disorder but a mild problem with the other (CSAT 1994).

A third model, called integrated treatment, combines elements of psychiatric and AOD treatment into one single program. Because a sufficient number of staff members are trained in both treatment approaches, diagnosis and treatment for both disorders can be conducted simultaneously, minimizing conflicts between the two approaches (Minkoff 1989). The integrated model is particularly suitable for comorbid patients who require relatively intensive or continuous psychiatric care. A limitation of the model is the tendency to undertreat addictive disorders and over-treat psychiatric disorders in patients seeking treatment for the psychiatric consequences of AOD disease (Minkoff 1989; Ries 1993).

The few studies that have assessed the outcome of integrated treatment have demonstrated effectiveness (Hoffman et al. 1993; Drake et al. 1993). One strength of this approach is its convenience to patients, thereby ensuring better compliance. In addition, integrated treatment enables most dual diagnosis patients to be “mainstreamed” into the basic addiction program, may reduce the patients’ sense of isolation, and may cost less than having patients treated in more than one location. In such integrated programs, many dual diagnosis patients can attend the same group or individual therapies as other patients and can participate in alcoholism treatments based on medications that help prevent relapse (e.g., naltrexone). In addition, certain antidepressants (e.g., desipramine) may decrease alcohol consumption among depressed alcoholics whose depression improves in response to medication (Mason 1996).

## Conclusions

Researchers have made great strides in understanding and treating persons with dual diagnoses. Improved diagnostic criteria are available and research has demonstrated that dually diagnosed patients have the best treatment outcomes only when both problems are addressed. The best type of treatment program is an integrated approach; although the treatments used are generally the same ones that are used for each disorder when treated separately, integration ensures that treatments will be coordinated for best effect.

One problem of dual diagnosis that is common to all current medical treatment is the lag between research advances and the practices of health maintenance organizations and other providers. Too often, single-disease management programs (i.e., “carve-outs”) dissociate addiction-focused treatment from medical, psychiatric, and other interventions that are needed by the patient, undermining the ability to develop integrated treatment models. Available data indicate that this dissociation is unwise, as discussed recently by Kessler and colleagues (1996). The development of cost-effective reimbursement practices must keep pace with developments in the effective treatment of people with dual disorders.

## Footnotes

1 For definitions of AOD and psychiatric disorders, see central glossary, p. 86.

2 Although the following discussion focuses primarily on alcohol-use disorders, most of these findings pertain to other drug-use disorders as well.

3 The relationships between antisocial personality disorder and alcohol are extremely complex and therefore cannot be fully discussed here. For further discussion, see Hesselbrock and colleagues (1986).

4 Withdrawal is a syndrome that begins 6 to 48 hours after cessation of alcohol consumption. It is characterized by tremors, elevated blood pressure, hallucinations, and, in severe cases, seizures.

5 Similarly, patients with schizophrenia or manic disorders may experience impulsive behavior or impaired judgment, leading to excessive involvement in AOD use along with other activities having potentially disastrous consequences (Woody et al. 1995; Goodwin and Jamison 1990).

## References

[b1-arhw-20-2-76] Allen JP, Columbus M (1995). Assessing Alcohol Problems: A Guide for Clinicians and Researchers.

[b2-arhw-20-2-76] American Psychiatric Association (1994). Diagnostic and Statistical Manual of Mental Disorders.

[b3-arhw-20-2-76] Cornelius JR, Salloum IM, Cornelius MD, Perel JM, Ehler JG, Jarrett PJ, Levin RL, Black A, Mann JJ (1995). Alcohol dependence. Preliminary report: Double-blind, placebo-controlled study of fluoxetine in depressed alcoholics. Psychopharmacology Bulletin.

[b4-arhw-20-2-76] Center for Substance Abuse Treatment (1994). Assessment and Treatment of Patients with Coexisting Mental Illness and Alcohol and Other Drug Abuse.

[b5-arhw-20-2-76] Drake RE, McHugo GJ, Noordsy DL (1993). Treatment of alcoholism among schizophrenic outpatients: 4-year outcomes. American Journal of Psychiatry.

[b6-arhw-20-2-76] Goodwin FK, Jamison KR (1990). Manic-Depressive Illness.

[b7-arhw-20-2-76] Grant BF, Towle LH (1990). Standardized diagnostic interviews for alcohol research. Alcohol Health & Research World.

[b8-arhw-20-2-76] Green VL (1996). The resurrection and the life. American Journal of Orthopsychiatry.

[b9-arhw-20-2-76] Helzer JE, Pryzbeck TR (1988). The co-occurrence of alcoholism with other psychiatric disorders in the general population and its impact on treatment. Journal of Studies on Alcohol.

[b10-arhw-20-2-76] Hesselbrock VM, Hesselbrock MN, Workman-Daniels KL (1986). Effect of major depression and antisocial personality on alcoholism: Course and motivational patterns. Journal of Studies on Alcohol.

[b11-arhw-20-2-76] Hoffman GW, DiRito DC, McGill EC (1993). Three-month follow-up of 28 dual diagnosis inpatients. American Journal of Drug and Alcohol Abuse.

[b12-arhw-20-2-76] Kadden RM, Kranzler HR, Rounsaville BJ (1995). Validity of the distinction between “substance-induced” and “independent” depression and anxiety disorders. American Journal on Addictions.

[b13-arhw-20-2-76] Kessler RC, Nelson CB, McGonagle KA, Edlund MJ, Frank RG, Leaf PJ (1996). The epidemiology of co-occurring addictive and mental disorders: Implications for prevention and service utilization. American Journal of Orthopsychiatry.

[b14-arhw-20-2-76] Khantzian EJ (1985). The self-medication hypothesis of addictive disorders: Focus on heroin and cocaine dependence. American Journal of Psychiatry.

[b15-arhw-20-2-76] Mason BJ, Kocsis JH, Ritvo EC, Cutter RB (1996). A double-blind, placebo-controlled trial of desipramine for primary alcohol dependence stratified on the presence or absence of major depression. Journal of the American Medical Association.

[b16-arhw-20-2-76] McLellan AT, Woody GE, O’Brien CP (1979). Development of psychiatric disorders in drug abusers. New England Journal of Medicine.

[b17-arhw-20-2-76] Merikangas KR, Gelernter CS (1990). Co-morbidity of alcoholism and depression. Psychiatric Clinics of North America.

[b18-arhw-20-2-76] Meyer RE (1989). Prospects for a rational pharmacotherapy of alcoholism. Journal of Clinical Psychiatry.

[b19-arhw-20-2-76] Miller NS (1994). Psychiatric comorbidity: Occurrence and treatment. Alcohol Health & Research World.

[b20-arhw-20-2-76] Minkoff K (1989). An integrated treatment model for dual diagnosis of psychosis and addiction. Hospital and Community Psychiatry.

[b21-arhw-20-2-76] National Institute on Alcohol Abuse and Alcoholism (1991). Alcohol Alert No. 14: Alcoholism and Co-occurring Disorders.

[b22-arhw-20-2-76] Raskin VD, Miller NS (1993). Epidemiology of the comorbidity of psychiatric and addictive disorders: A critical review. Journal of Addictive Diseases.

[b23-arhw-20-2-76] Regier DA, Farmer ME, Rae DS, Locke BZ, Keith SJ, Judd LL, Goodwin FK (1990). Comorbidity of mental disorders with alcohol and other drug abuse. Journal of the American Medical Association.

[b24-arhw-20-2-76] Ries R (1993). Clinical treatment matching models for dually diagnosed patients. Psychiatric Clinics of North America.

[b25-arhw-20-2-76] Rounsaville BJ, Glazer W, Wilber CH, Weissman MM, Kleber HD (1983). Short-term interpersonal psychotherapy in methadone maintained opiate addicts. Archives of General Psychiatry.

[b26-arhw-20-2-76] Roy A, DeJong J, Lamparski D, George T, Linnoila M (1991). Depression among alcoholics. Archives of General Psychiatry.

[b27-arhw-20-2-76] Schuckit MA (1983). Alcohol patients with secondary depression. American Journal of Psychiatry.

[b28-arhw-20-2-76] Schuckit MA (1985). The clinical implications of primary diagnostic groups among alcoholics. Archives of General Psychiatry.

[b29-arhw-20-2-76] Schuckit MA (1986). Genetic and clinical implications of alcoholism and affective disorder. American Journal of Psychiatry.

[b30-arhw-20-2-76] Schuckit MA, Monteiro MG (1988). Alcoholism, anxiety and depression. British Journal of Addiction.

[b31-arhw-20-2-76] Winokur G, Coryell W, Akiskal HS, Maser JD, Keller MB, Endicott J, Mueller T (1995). Alcoholism in manic-depressive (bipolar) illness: Familial illness, course of illness, and the primary-secondary distinction. American Journal of Psychiatry.

[b32-arhw-20-2-76] Woody GE, McLellan AT, Bedrick J, Oldham JM, Riba MB (1995). Dual Diagnosis. Review of Psychiatry.

